# Penile abscess and necrotizing fasciitis secondary to neglected false penile fracture

**DOI:** 10.4103/0974-7796.65111

**Published:** 2010

**Authors:** Reshaid Abdullah Al-Reshaid, Khaled Madbouly, Abdullah Al-Jasser

**Affiliations:** Urology Division, Surgery Department, Security Forces Hospital, Ryiadh, Kingdom of Saudi Arabia

**Keywords:** Corpora cavernosa, penile abscess, penile fracture, tunica albuginea

## Abstract

Penile infection and abscess formation have been described in association with priapism, cavernosography, intracavernosal injection therapy, trauma and penile prosthesis. We report a case of penile abscess and necrotizing fasciitis of penile skin in a 37-year-old male, presented 3 weeks after neglected false penile fracture.

## INTRODUCTION

The few cases of purulent cavernositis or corporeal infection in the literature have generally resulted as a complication of trauma, intracavernous injection therapy, priapism, cavernosography or foreign body.[1] Also, penile fracture is an uncommon, although well-described, urologic emergency. It refers to rupture of the tunica albuginea of the corpus cavernosum during sexual activity.[2] Clinically, most patients report penile pain associated with a snap sound and immediate detumescence. Penile fracture mimics, or false fractures, have been infrequently described.[2-4] They are difficult, or impossible, to distinguish clinically from true penile fracture.

We report a rare case of penile abscess secondary to conservative management of false penile fracture. The case was further complicated by development of necrotizing fasciitis of the penile shaft.

## CASE REPORT

A 37-year-old afebrile male presented to the emergency department with swelling, change of skin color and evident pus discharge from the proximal penile shaft dorsally to the left side. The swelling complicated severe pain and penile detumescence but with no snap sound during sexual intercourse before 3 weeks. No more erections were reported thereafter. The patient denied any history of urethral discharge, intracorporeal therapy or instrumentation.

**Figure 1:**
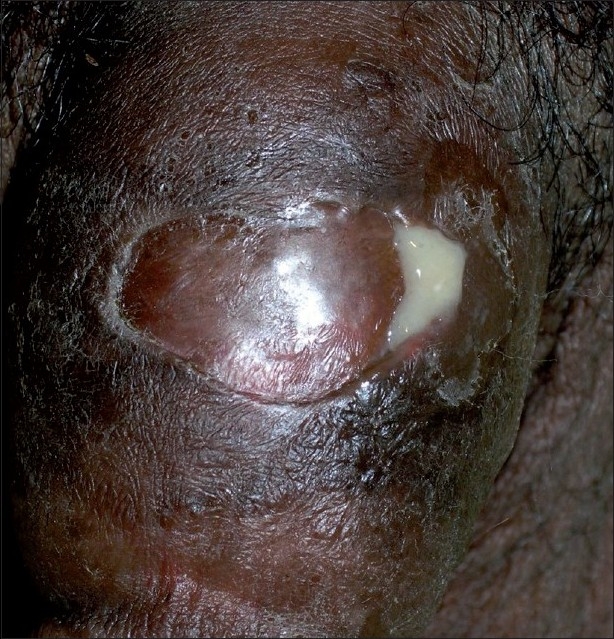
Penile abscess with evident pus discharge

Physical examination revealed edema, redness, ulceration and discharge of thick, yellowish pus from the proximal penile shaft [Figure 1]. The external urethral meatus showed no urethral discharge. No rolling sign or corporal defects could be detected. Both testes and epididymides were palpably normal. Inguinal lymph nodes were not enlarged. Rectal examination was unremarkable.

Random blood sugar and white cell count were within normal. Urine analysis and culture showed no evidence of infection. HIV serology and urine culture for tuberculosis were negative.

**Figure 2:**
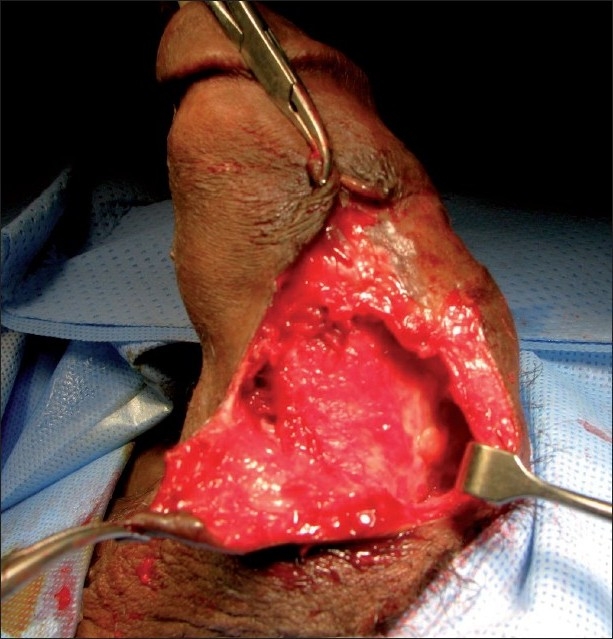
Abscess drainage and debridement down to the intact cavernosal tunica albuginea

**Figure 3:**
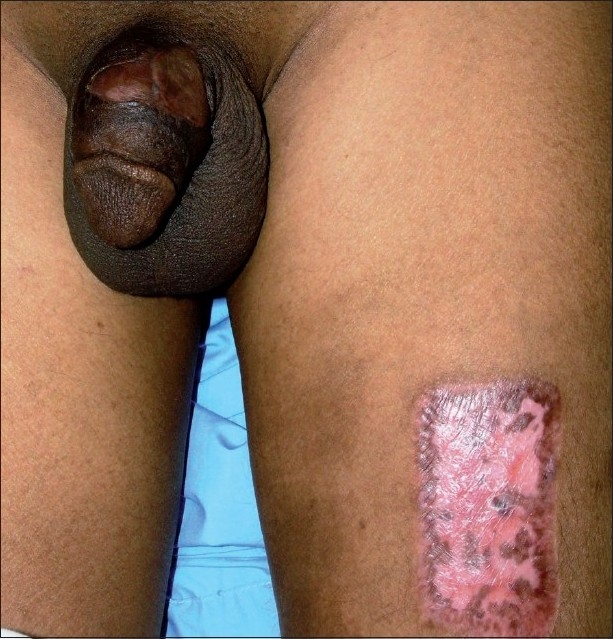
A viable graft at follow up

Under spinal anesthesia the penile abscess was incised, pus was drained and a 4 × 5 cm area of necrotic skin was excised down to the intact cavernosal tunica albuginea [Figure 2]. Irrigation with saline and povidone iodine was carried out. Further debridement was required after 48 hours. The patient was kept on IV ciprofloxacine and daily dressing for 2 weeks when a full thickness skin graft was taken from the left thigh and applied to the penile shaft while artificially erected. The dressing was removed after 1 week and the patient was sent home with a viable graft. Follow up after 2 weeks revealed a viable graft [Figure 3], normal voiding and normal morning erection with no deviation, pain or shortening.

## DISCUSSION

Penile abscesses are rare and can develop after trauma, as a complication of cavernosography, as an unusual presentation of gonorrhea, after intracorporeal injections or penile prosthesis insertion.[1] It can usually be managed by drainage and antibiotic therapy.[1,5] Our patient had no history of diabetes, immunodeficiency, urethral discharge, trauma or penile manipulations that could explain abscess formation. Secondary infection of the penile hematoma formed as a result of penile fracture is the most plausible mechanism.

Penile fracture is a well-described entity that refers to rupture of the tunica albuginea of the corpus cavernosum during sexual activity.[2] Although the patient's history was clearly suggestive of penile fracture, on exploration, the corpus cavernosum and its tunica albuginea were intact with no thickening, plaque or curvature formation suggesting a false penile fracture.

False fractures of the penis have been reported previously where both corpora were found to be intact and the hematoma was noted within the Dartos fascia.[2] Clinically, absence of the snap and/or gradual detumescence are suggestive without being specific.[2,3] The utility of imaging studies in the setting of the suspected penile fracture is controversial. Ultrasonography and more recently magnetic resonance imaging (MRI) have been recommended when the diagnosis is uncertain.[6,7]

Immediate surgical intervention with evacuation of hematoma and repair of the ruptured tunica albuginea is widely accepted as the treatment of choice for penile fracture.[8,9] Conservative management of penile fracture results in penile curvature in more than 10% of patients, or debilitating plaques in 25–30%, and significantly longer hospitalization times and recovery.[9] On exploration, Buck's fascia was eroded by the abscess with development of necrotizing fasciitis. Abscess drainage, antibiotic therapy and debridement resulted in complete eradication of infection. A full thickness skin graft successfully replaced the lost penile tissues. At follow up, the patient reported good erection with no deviation.

Our case scenario still recommends immediate penile exploration in patients with suspected penile fracture.
